# Mitochondrial Activity Regulates Human T Helper 17 Differentiation and Function

**DOI:** 10.1111/imm.70037

**Published:** 2025-09-17

**Authors:** Xinlai Chen, Theodoros Ioannis Papadimitriou, Anne H. G. van Essen, Britt van Brunschot, Monique M. Helsen, Annet Sloetjes, Elly L. Vitters, Werner J. H. Koopman, Peter M. van der Kraan, Arjan P. M. van Caam, Marije I. Koenders

**Affiliations:** ^1^ Department of Rheumatology and Laboratory of Experimental Rheumatology Radboudumc Nijmegen the Netherlands; ^2^ Department of Laboratory Medicine – Medical Immunology Radboudumc Nijmegen the Netherlands; ^3^ Department of Pediatrics, Amalia Children's Hospital Radboudumc Nijmegen the Netherlands; ^4^ Radboud Center for Mitochondrial Medicine Radboudumc Nijmegen the Netherlands; ^5^ Human and Animal Physiology Wageningen University Wageningen the Netherlands

**Keywords:** mitochondrial activity, T cell exhaustion, T cell metabolism, Th17 cells, Th17 differentiation

## Abstract

Immunometabolism plays a pivotal role in T cell fate decisions, yet its specific contribution to human Th17 differentiation remains incompletely understood. Th17 cells, a subset of CD4^+^ T cells, are central to autoimmune pathogenesis through their secretion of pro‐inflammatory cytokines. Elucidating the metabolic drivers of Th17 differentiation may reveal novel therapeutic targets. We investigated the role of mitochondrial activity in Th17 differentiation using an in vitro model with naïve human CD4^+^ T cells. Single‐cell metabolic profiling and functional assays were used to characterise metabolic changes during differentiation. Th17 cells exhibited a hyperpolarised mitochondrial membrane potential (ΔΨ) compared to non‐Th17 cells. Hyperpolarised ΔΨ cells displayed increased metabolic activity and enhanced differentiation capacity. Metabolic profiling at 48 h revealed an early reliance on glycolysis, followed by a shift toward increased dependence on oxidative phosphorylation (OXPHOS) by 96 h. Gene expression analysis indicated early upregulation of *TEFM*, a mitochondrial transcription regulator, at 48 h. By 96 h, ΔΨ hyperpolarised cells exhibited a downregulation of *DRP1* and *MFN2*, genes responsible for mitochondrial fission and fusion. Functionally, ΔΨ hyperpolarised cells expressed elevated activation markers (CD69, CD25) but also showed increased exhaustion markers (TIGIT, PD‐1), indicating a link between high metabolic activity and exhaustion. Additionally, these cells triggered weaker NF‐κB and AP‐1 signalling and secreted lower levels of effector molecules (IFN‐γ, Granzyme B) than ΔΨ depolarised cells. In conclusion, mitochondrial activity critically shapes Th17 differentiation. Although hyperpolarised ΔΨ cells exhibit greater activation, they are more prone to exhaustion and reduced effector function. These findings offer insights into Th17 metabolic regulation and its therapeutic potential in autoimmune diseases.

## Graphical abstract

Naïve CD4^+^ T cells differentiating into RORγt^+^IL‐17A^+^ Th17 cells are characterised by a shift from glycolysis to oxidative phosphorylation. Early‐stage cells favour glycolysis, whereas later‐stage cells with hyperpolarised mitochondrial membrane potential (ΔΨ) rely more on oxidative phosphorylation, show increased activation, and differentiate more readily into Th17 cells, but are prone to functional exhaustion and reduced secretion of effector molecules (IFN‐γ, Granzyme B). In contrast, depolarised ΔΨ cells rely more on glycolysis and show reduced Th17 polarisation. This paper describes how mitochondrial activity shapes functional heterogeneity in Th17 differentiation and highlights its potential as a therapeutic target in autoimmune diseases.

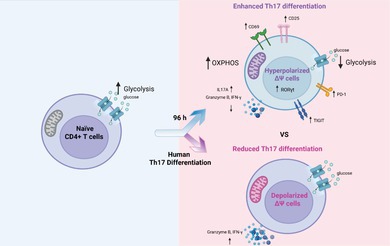

## 1 Introduction

T helper 17 (Th17) cells play a pivotal role in host defence against extracellular pathogens, including bacteria and fungi [1, 2]. These cells originate from naive CD4^+^ T cell precursors [3, 4] and are characterised by the expression of the transcription factor RAR‐related orphan receptor C (RORC) and the production of interleukin (IL)‐17A [5]. Beyond their protective function, Th17 cells are critically involved in the pathogenesis of various autoimmune diseases, such as inflammatory bowel disease [6, 7], spondyloarthritis [8], psoriatic arthritis [9] and rheumatoid arthritis (RA) [10]. Targeting IL‐17A has demonstrated significant therapeutic efficacy in reducing disease activity and improving clinical outcomes in these conditions [11, 12].

Understanding the mechanisms governing Th17 cell differentiation is essential for devising strategies to prevent the emergence and expansion of pathogenic Th17 cells during the early stages of autoimmune disease. Early intervention is considered critical in achieving disease remission. However, the differentiation of Th17 cells—particularly in humans—remains incompletely understood. Further insights into these mechanisms could help restore immunological homeostasis by preventing the formation of pathogenic Th17 cells [13].

In addition to the three classical signals that drive CD4^+^ T cell differentiation—T cell receptor activation, co‐stimulation, and the cytokine milieu—recent studies emphasise the crucial role of cell metabolism in determining T cell fate and function [14, 15]. Metabolic reprogramming, particularly the interplay between glycolysis, fatty acid oxidation (FAO), and mitochondrial oxidative phosphorylation (OXPHOS), is essential for the differentiation of naïve T cells into the Th17 phenotype. For instance, in murine models of central nervous system autoimmunity, the metabolic switch between glycolysis and FAO directly guides Th17 cells toward a more pathogenic state, characterised by the production of IFN‐γ [16]. Disrupting OXPHOS in these polyfunctional Th17 cells alters their gene expression, causing them to adopt a profile more akin to non‐pathogenic Th17 cells [17]. Furthermore, OXPHOS has been shown to enhance the resistance of effector Th17 cells to apoptosis, which may contribute to their prolonged persistence in vivo and the potential to acquire memory‐like properties [18].

However, the extent to which these metabolic programmes regulate Th17 differentiation in human cells remains unclear. Given that differentiating human Th17 cells from naive T cells in vitro remains a significant challenge, current knowledge on the metabolic factors regulating Th17 development is largely dependent on murine studies. Thus, whether mitochondrial metabolism similarly shapes the differentiation and function of human Th17 cells remains an open question.

In this study, we directly addressed this gap by investigating how mitochondrial activity influences the differentiation of human naive CD4^+^ T cells into effector Th17 cells. By developing an in vitro human Th17 differentiation model and utilising single‐cell metabolic profiling techniques, we explored the role of mitochondrial function in shaping Th17 formation. Our findings provide new insights into the energy metabolic regulation of human Th17 cells and highlight potential therapeutic opportunities for targeting mitochondrial function in autoimmune disease.

## 2 Results

### 2.1 Th17 Cells Exhibit Hyperpolarised Mitochondrial Membrane Potential Compared to Non‐Th17 Cells

**FIGURE 1:**
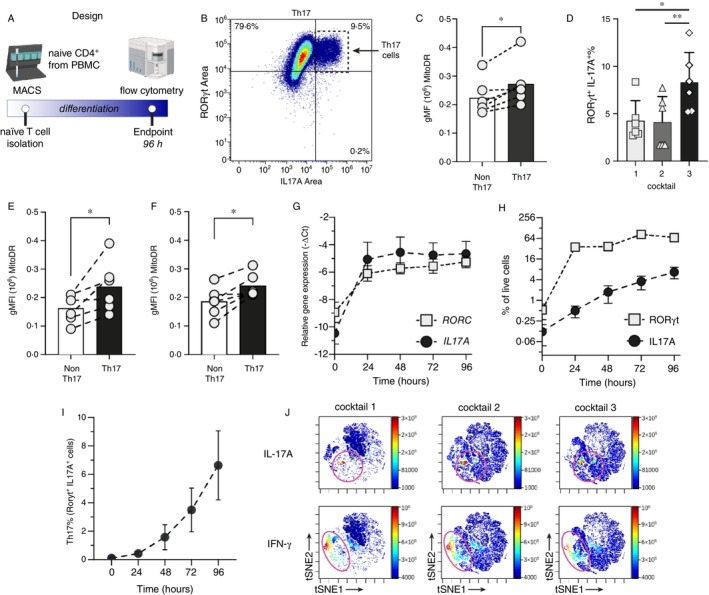
Th17 Cells exhibit higher mitochondrial activity than non‐Th17 cells. (A) Schematic representation of the in vitro Th17 differentiation process. (B) Representative flow cytometry shows Th17 cells (RORγt^+^ IL‐17A^+^) generated after 96 h under optimised conditions. (C) Mitochondrial membrane potential (ΔΨ), assessed via MitoTracker Deep Red (MDR) geometric mean fluorescence intensity (gMFI), is significantly higher in Th17 cells compared to non‐Th17 counterparts (*n* = 6, *p* = 0.0117). (D) Th17 cell frequency is significantly increased under the optimised polarisation cocktail (cocktail 3) compared to cocktails 1 and 2 (*n* = 6; cocktail 1 versus 3, *p* = 0.0312; cocktail 2 vs. 3, *p* = 0.0079). (E, F) MDR gMFI is also elevated in Th17 cells versus non‐Th17 cells under cocktail 1 (*p* = 0.0363) and cocktail 2 (*p* = 0.0129), respectively. (G) Mean RORC and IL17 mRNA expression over time during in vitro Th17 differentiation (*n* = 6). (H) Mean RORγt and IL‐17A protein production over time during in vitro Th17 differentiation (*n* = 6). (I) Kinetics of Th17 cell differentiation over time as determined by flow cytometry (*n* = 6). (J) viSNE analysis of Th17 polarisation, comparing cytokine production across three Th17 polarisation cocktails with equal event counts. The optimised cocktail yields the highest IL‐17A production with reduced IFN‐γ levels. Data for panels G, H, and I are presented as mean ± SD. Error bars represent standard deviation.

Naive CD4^+^ T cells were differentiated into Th17 cells over a 96‐h in vitro culture under Th17‐polarising conditions (Figure 1A). Th17 cells, identified by flow cytometry as RORγt^+^IL‐17A^+^ populations (Figure 1B; gating strategy shown in Figure S1), were distinguished from non‐Th17 cells lacking these markers. Mitochondrial membrane potential (ΔΨ), an indicator of mitochondrial bioenergetic status, was assessed using MitoTracker Deep Red (MDR) staining and quantified as the geometric mean of cellular fluorescence intensity (gMFI). Higher MDR accumulation, consistent with a hyperpolarised ΔΨ, was observed in Th17 cells compared to non‐Th17 cells (Figure 1C).

To validate the reproducibility of this observation, we compared three Th17 polarisation protocols (cocktail 1, cocktail 2, and our optimised cocktail 3; Table S1), reflecting common variations used for in vitro human Th17 differentiation [19, 20]. Although the field employs multiple differentiation protocols, we sought to determine whether the mitochondrial phenotype—specifically increased MDR accumulation—was consistent across different conditions. All three cocktails successfully induced Th17 differentiation, with our optimised cocktail achieving the highest yield of IL‐17A^+^ Th17 cells (Figure 1D). Across all conditions (*n* = 6), Th17 cells consistently exhibited higher MDR staining intensity than non‐Th17 cells (Figure 1E,F), supporting a robust association between mitochondrial hyperpolarisation and Th17 identity.

Early transcriptional analysis revealed upregulation of *RORC* and *IL17A* mRNA as early as 24 h (Figure 1G), although IL‐17A protein expression was not detected until 48–72 h (Figure 1H). Initiation of Th17 polarisation was detectable at 24 h but became more robust from 48 h onward (Figure 1I). Based on these kinetics, subsequent analyses of mitochondrial activity focused on 48‐h and 96‐h time points to capture early and late phases of Th17 development.

To further assess the specificity of Th17 differentiation across cocktails, we evaluated the expression of the effector cytokine IFN‐γ alongside IL‐17A by intracellular flow cytometry. Our optimised cocktail (cocktail 3) produced the highest proportion of IL‐17A^+^ cells with reduced IFN‐γ production, whereas cocktails 1 and 2 predominantly induced IFN‐γ^+^ cells (Figure 1J). All conditions were analysed using equal numbers of input cells, confirming that observed differences reflected true differences in Th17 yield and cytokine profile.

To characterise T helper subset heterogeneity, we further analysed the intracellular protein expression of lineage‐defining transcription factors, including T‐bet, FOXP3, and GATA3, alongside their signature cytokines (IL‐4, IFN‐γ, and IL‐10), by multicolour flow cytometry. Our optimised cocktail generated the lowest frequency of non‐Th17 T helper subsets, as reflected by a reduced frequency of non‐Th17 markers (Figure S2).

Given these findings, we next sought to determine whether the observed differences in mitochondrial membrane potential merely correlate with Th17 differentiation or actively influence Th17 fate. To address this, we sorted cells based on MDR staining intensity at defined time points and performed functional analyses to investigate the impact of mitochondrial activity on Th17 commitment and metabolic reprogramming.

### 2.2 Hyperpolarized ΔΨ Cells Exhibit Increased Metabolic Activity and Enhanced Th17 Differentiation

**FIGURE 2:**
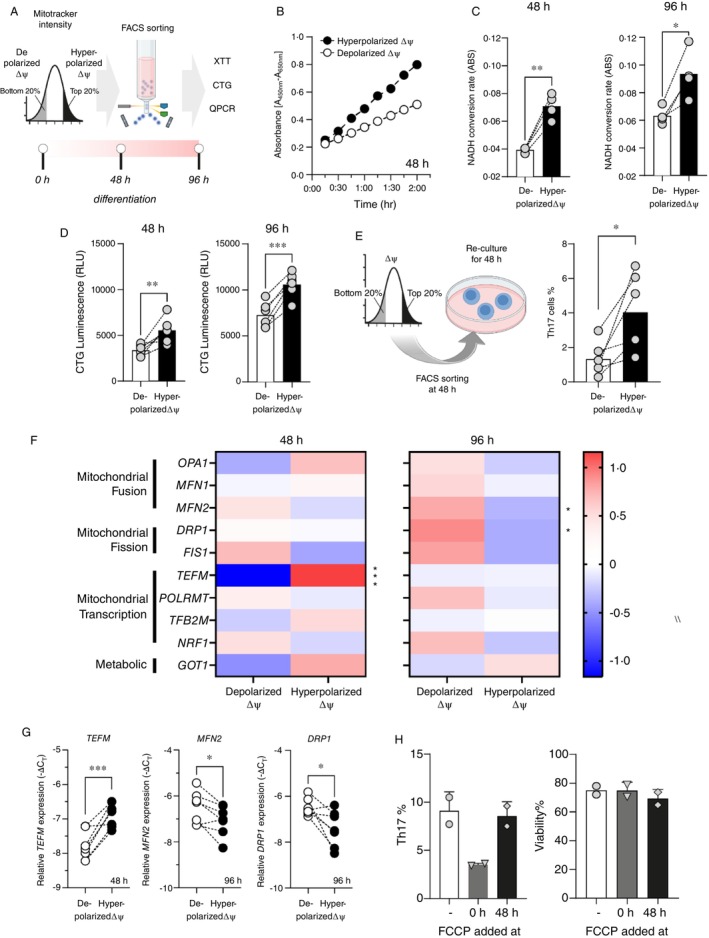
Hyperpolarized ΔΨ cells exhibit increased metabolic activity and enhanced Th17 differentiation. (A) A schematic illustrates the sorting strategy of hyperpolarized ΔΨ cells and depolarized ΔΨ cells CD4^+^ T cells at 48 and 96 h of Th17 differentiation for further analysis. (B) Representative XTT absorbance data, indicating NADH conversion hyperpolarized ΔΨ cells and depolarized ΔΨ cells sorted by FACS at 48 h. Absorbance was measured every 15 min over a 2‐h period using a colorimetric assay. (C) XTT analysis (*n* = 6) revealed a significant difference in NADH conversion rates between hyperpolarized ΔΨ cells and depolarized ΔΨ cells at both 48 h (*n* = 6 unique donors, *p* = 0.0017) and 96 h (*n* = 6 unique donors, *p* = 0.0107). (D) Total ATP content, measured by CellTiter‐Glo (CTG), in hyperpolarized ΔΨ cells and depolarized ΔΨ cells at both 48 h (*n* = 6 unique donors, *p* = 0.0060) and 96 h (*n* = 6 unique donors, *p* = 0.0017). Luminescence (RLU) was used as a proxy for metabolic activity. (E) Th17 differentiation was assessed at Day 4 after re‐seeding sorted hyperpolarized ΔΨ cells and depolarized ΔΨ cells into the Th17 polarisation cocktail at 48 h (*n* = 6 unique donors, *p* = 0.0219). (F) Gene expression analysis of mitochondrial dynamics‐related genes in hyperpolarized ΔΨ cells and depolarized ΔΨ cells at 48 and 96 h, visualised in a heatmap. Gene expression values were normalised using Z‐scores for better comparison across samples. (G) TEFM mRNA expression in hyperpolarized ΔΨ cells and depolarized ΔΨ cells at 48 h (*p* = 0.0071); MFN2 and DRP1 expression in ΔΨm high and ΔΨm low cells at 96 h (*n* = 6 unique donors; DRP1 *p* = 0.0461; MFN2 *p* = 0.0391). (H) FCCP (500 nM) was added at 0 or 48 h during a 96‐h culture period. Th17 yield and cell viability were measured by flow cytometry. Data represent mean ± SD from two donors. Statistical significance was determined using paired t‐tests or Wilcoxon tests where appropriate.

To directly assess the relationship between mitochondrial activity and human Th17 differentiation, we sorted hyperpolarised and depolarised ΔΨ CD4^+^ T cells at 48 and 96 h and evaluated their metabolic profiles and differentiation capacity (Figure 2A). ΔΨ was determined by MitoTracker Deep Red (MDR) staining. To assess metabolic activity, we measured NADH conversion rates over a 2‐h period using the XTT assay, with readings taken every 15 min. Hyperpolarised ΔΨ cells consistently exhibited higher NADH conversion compared to depolarised ΔΨ cells at both 48 and 96 h (Figure 2B,C), indicating greater metabolic activity. These results were consistent across three independent experiments, each involving two donors.

Total ATP content was then assessed using the CellTiter‐Glo (CTG) assay, which quantifies total cellular ATP levels based on luminescence. Hyperpolarized ΔΨ cells displayed significantly higher ATP content than depolarized ΔΨ cells at both time points (Figure 2D). All measurements were performed on highly viable cells, and viability was comparable across groups.

Based on these metabolic differences, we re‐sorted hyperpolarized and depolarized ΔΨ cells at 48 h and re‐seeded them into Th17‐polarising conditions for an additional 48 h. Following extended culture, hyperpolarized ΔΨ cells exhibited significantly enhanced Th17 differentiation compared to depolarized ΔΨ cells (Figure 2E). These findings suggest that elevated metabolic activity is associated with an increased capacity for Th17 lineage commitment.

Given the importance of mitochondrial morphology and structure in T cell function, we next assessed the expression of genes involved in mitochondrial fusion (*OPA1*, *MFN1*, *MFN2*), fission (*DRP1*, *FIS1*), transcription (*TEFM*, *POLRMT*, *TFB2M*, *NRF1*), and metabolism (*GOT1*, which has been linked to Th17 differentiation [21]) at 48 and 96 h (Figure 2F). Gene expression was normalised to housekeeping genes using the −ΔCt method. At 48 h, *TEFM* was significantly upregulated in hyperpolarized ΔΨ cells compared to depolarized cells (Figure 2G), suggesting enhanced mitochondrial transcriptional activity during early Th17 differentiation. By 96 h, hyperpolarized ΔΨ cells exhibited downregulation of *DRP1* and *MFN2* (Figure 2G), suggesting dynamic regulation of mitochondrial morphology as differentiation progressed.

To further investigate the functional role of mitochondrial activity, we used the mitochondrial uncoupler carbonyl cyanide‐p‐trifluoromethoxy phenylhydrazone (FCCP) to disrupt ΔΨ. FCCP was applied at a final concentration of approximately 500 nM. As expected, FCCP treatment reduced MDR fluorescence intensity (data not shown). When FCCP was added at the beginning of culture (0 h), Th17 differentiation was significantly impaired; however, when FCCP was added at 48 h, no clear inhibition was observed (Figure 2H). These results suggest that mitochondrial activity is particularly critical during the early stages of Th17 polarisation.

### 2.3 ΔΨ High T Cells Favour Glucose Uptake and Glycolysis Over FAO and AAO Cap and OXPHOS During Th17 Differentiation

**FIGURE 3:**
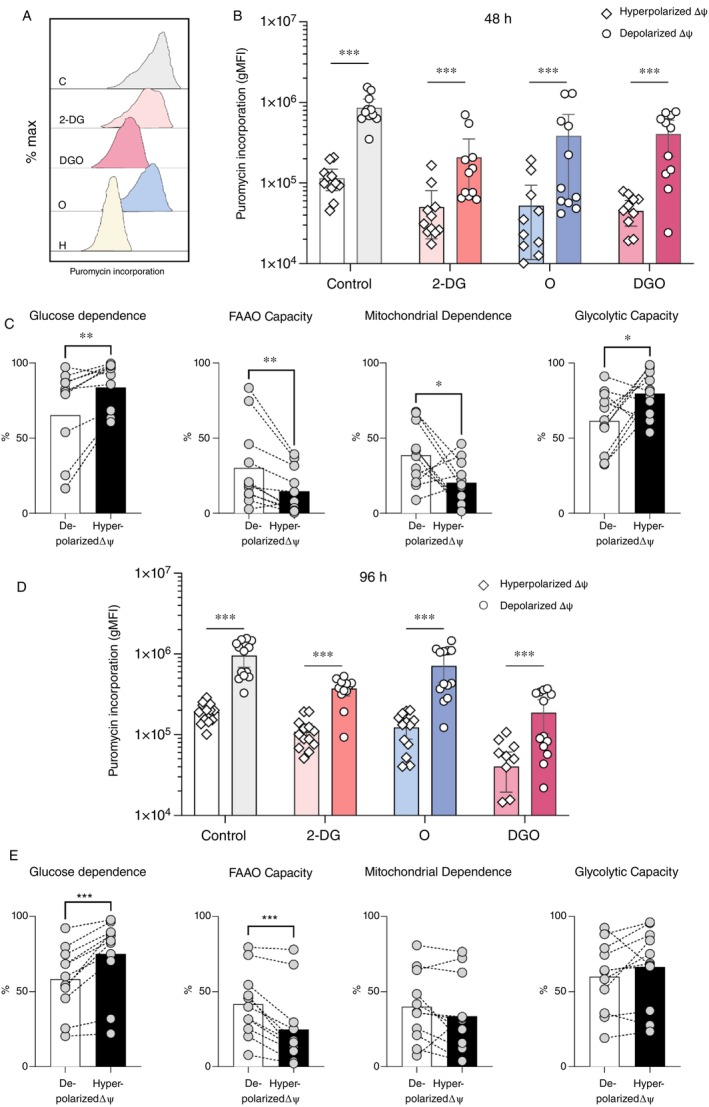
Metabolic profiles of hyperpolarized ΔΨ cells and depolarized ΔΨ cells during Th17 differentiation. (A) Representative example of puromycin incorporation (gMFI) in hyperpolarized ΔΨ cells and depolarized ΔΨ cells, analysed using the SCENITH workflow, illustrating metabolic dependencies during Th17 differentiation. (B) Puromycin incorporation (MFI) at 48 h in hyperpolarized ΔΨ cells and depolarized ΔΨ cells under control, 2‐DG, oligomycin, and DGO conditions, showing significantly higher incorporation in hyperpolarized ΔΨ cells (*n* = 11 unique donors; control *p* < 0.0001, 2‐DG *p* = 0.001, oligomycin *p* = 0.0001, DGO *p* = 0.001). (C) Comparative metabolic profiles of hyperpolarized ΔΨ cells and depolarized ΔΨ cells at 48 and 96 h reveal significant differences in glucose dependence (*p* = 0.0029), fatty acid and amino acid oxidation (FAAO) capacity (*p* = 0.0029), mitochondrial dependence (*p* = 0.0473), and glycolytic capacity (*p* = 0.0473) across 11 unique donors. (D) Puromycin incorporation at 96 h shows consistent differences between hyperpolarized ΔΨ cells and depolarized ΔΨ cells (*n* = 11; control *p* < 0.0001, 2‐DG *p* < 0.0001, oligomycin *p* = 0.0001, DGO *p* < 0.0001). (E) Metabolic profiles at 96 h demonstrate significant differences in glucose dependence (*p* = 0.001) and FAAO capacity (*p* = 0.001). Statistical comparisons were performed using paired *t* tests or Wilcoxon matched‐pairs tests, as appropriate.

To further characterise the metabolic differences between hyperpolarized ΔΨ and depolarized ΔΨ cells during Th17 differentiation, we employed the SCENITH technique [22] at 48 and 96 h. SCENITH quantifies protein synthesis under specific metabolic inhibition conditions, allowing inference of pathway utilisation and metabolic dependencies (Figure 3A). This method measures puromycin incorporation into nascent proteins as a direct proxy for available ATP, following treatment with specific inhibitors: 2‐deoxy‐D‐glucose (2‐DG) to block glycolysis, oligomycin (O) to inhibit oxidative phosphorylation (OXPHOS), and their combination (DGO). Harringtonine, a general inhibitor of translation, served as a positive control. These conditions allow assessment of glycolytic, mitochondrial, fatty acid oxidation (FAO), and amino acid oxidation (AAO) capacities.

At 48 h, hyperpolarized ΔΨ cells exhibited significantly higher geometric mean fluorescence intensity (gMFI) of puromycin incorporation compared to depolarized ΔΨ cells across all SCENITH conditions (control, 2‐DG, oligomycin, and DGO) (Figure 3B). Of note, only a subset of hyperpolarized ΔΨ cells corresponds to Th17 cells, as sorting directly based on Th17 markers was technically challenging under live‐cell conditions. By 96 h, hyperpolarized ΔΨ cells maintained higher gMFI values under control, 2‐DG, oligomycin, and DGO treatments (Figure 3D), indicating sustained overall metabolic activity reflected by elevated protein synthesis.

Further analysis of metabolic pathway contributions revealed distinct metabolic dependencies between hyperpolarized and depolarized ΔΨ cells during Th17 polarisation. At 48 h, hyperpolarized ΔΨ cells displayed significantly greater glucose dependence and glycolytic capacity compared to depolarized ΔΨ cells. Conversely, they exhibited lower reliance on FAO and AAO capacities, as well as reduced mitochondrial dependency, suggesting a preferential engagement of glycolysis for energy generation at this stage (Figure 3C).

By 96 h, these metabolic distinctions persisted in terms of glucose dependence and reduced FAO and AAO capacities, with hyperpolarized ΔΨ cells continuing to favour glucose metabolism. However, no significant differences were observed in glycolytic capacity or mitochondrial dependence between the two groups at this later time point (Figure 3E). This may suggest that, over time, hyperpolarized ΔΨ cells begin to adjust their metabolic reliance, although direct evidence of a functional metabolic shift would require additional validation, such as lactate production assays. Together, these findings suggest that hyperpolarized ΔΨ T cells primarily rely on glycolysis over FAO, AAO, and OXPHOS during early Th17 polarisation, although whether these metabolic distinctions translate into functional differences remains to be fully determined.

### 2.4 Mitochondrial Activity Defines Activation, Signalling, and Functional Heterogeneity of Th17 Cells

**FIGURE 4:**
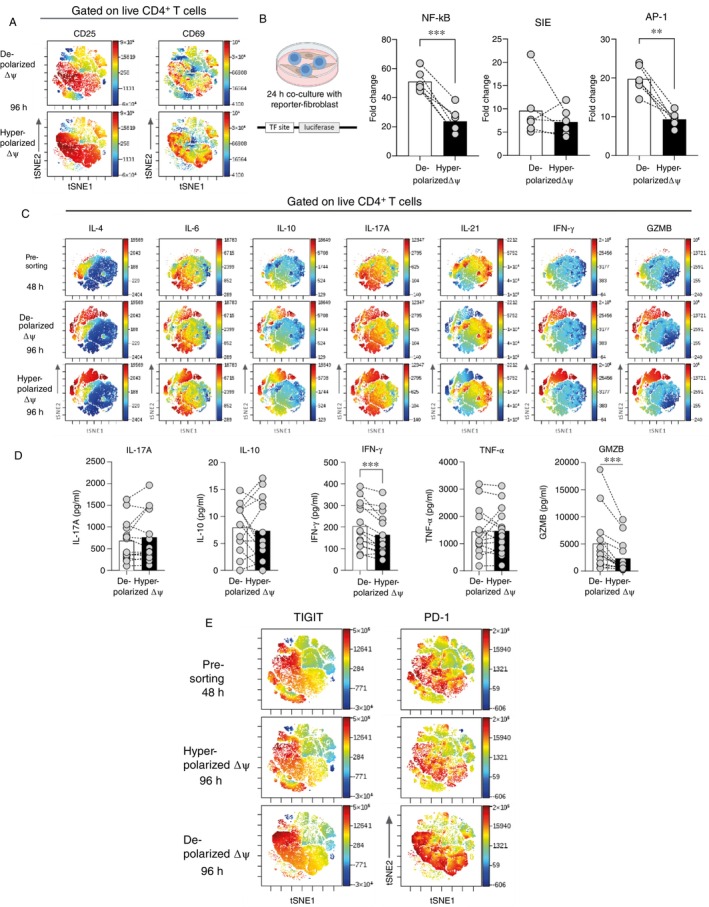
Hyperpolarized ΔΨ cells exhibit enhanced activation but reduced cytokine secretion, weaker signalling activation, and elevated exhaustion. (A) Assessment of CD25 and CD69 expression in hyperpolarized ΔΨ cells and depolarized ΔΨ cells at 96 h (*n* = 6 unique donors). (B) Activation of NF‐κB (*p* = 0.0003), SIE, and AP‐1 (*p* = 0.0004) signalling pathways in sorted hyperpolarized ΔΨ cells and depolarized ΔΨ cells. Reporter activity for NF‐κB, JAK–STAT, and AP‐1 pathways was normalised to the activity observed in negative control conditions. Data represent normalised fold changes relative to the negative control (*n* = 6 unique donors). (C) Cytokine production potential was measured after a 4‐h PMA/ionomycin stimulation with Golgi inhibition to assess intracellular cytokine levels (*n* = 6 unique donors). (D) Secreted cytokine levels were quantified from culture supernatants using Luminex. Cytokine secretion of IFN‐γ (*p* = 0.0007) and GZMB (*p* = 0.0009) was significantly lower in hyperpolarized ΔΨ cells. No significant differences were observed in IL‐17A, IL‐10, or TNF‐α secretion. (E) Exhaustion markers (e.g., TIGIT, PD‐1) were evaluated on day 4 in sorted cells (*n* = 6 unique donors). Data for panels A, C, and E were processed and analysed using vi‐SNE (Cellular Interaction Tuning by Recursive Sampling) in Cytobank for further interpretation of activation, exhaustion signatures, and cytokine production.

To determine whether mitochondrial activity influences Th17 cell function, we examined activation status, signalling potential, and cytokine production capacity in hyperpolarized and depolarized ΔΨ T cells. Cells were sorted at 48 h using FACS and cultured for an additional 48 h in our optimised Th17 polarisation cocktail (cocktail 3). By 96 h, hyperpolarized ΔΨ cells exhibited a significantly higher Th17 yield (Figure 2E) and increased expression of the activation markers CD25 and CD69, compared to depolarized ΔΨ cells (Figure 4A), indicating a heightened activation state.

Given that T cell activation can influence downstream signalling, we examined how hyperpolarized and depolarized ΔΨ cells engaged key pathways. We co‐cultured them with pathway‐specific reporter fibroblast lines at a 1:1 ratio for 24 h. These fibroblasts were engineered to report activation of NF‐κB, MAPK (AP‐1), and JAK–STAT signalling pathways. Notably, depolarized ΔΨ cells induced significantly higher activation of NF‐κB and AP‐1 compared to hyperpolarized ΔΨ cells (Figure 4B), suggesting that cells with lower mitochondrial membrane potential may more effectively trigger inflammatory signalling. No significant differences were observed for STAT‐mediated (SIE) reporter activity between the two groups, highlighting the specificity of the differences observed.

To further investigate functional consequences linked to mitochondrial activity, we assessed intracellular cytokine production. At 48 h, both populations exhibited minimal cytokine expression. However, after an additional 48 h of culture (96 h total), hyperpolarized ΔΨ cells displayed significantly higher expression MFI levels of IL‐4, IL‐6, IL‐10, IL‐17A, IFN‐γ, IL‐21, and granzyme B (GZMB) compared to depolarized ΔΨ cells (Figure 4C). These findings indicate that hyperpolarized ΔΨ cells are primed for effector cytokine production following Th17 differentiation. Notably, cytokine expression was measured after 4 h of PMA/ionomycin stimulation in the presence of a Golgi transport inhibitor to reflect production capacity rather than basal secretion.

To complement these findings, we assessed cytokine secretion by performing a Luminex assay on culture supernatants collected at 96 h (Figure 4D). Depolarized ΔΨ cells secreted significantly higher levels of IFN‐γ and GZMB compared to hyperpolarized ΔΨ cells, whereas secretion of IL‐17A, IL‐10, and TNF‐α showed no significant differences. This discrepancy between intracellular cytokine expression and secreted cytokine levels suggests functional suppression in hyperpolarized ΔΨ cells. We hypothesised that this suppression might be linked to exhaustion. Consistent with this, hyperpolarized ΔΨ cells exhibited significantly higher expression of the exhaustion markers PD‐1 and TIGIT at 96 h compared to depolarized ΔΨ cells (Figure 4E).

Together, these findings suggest that mitochondrial activity influences not only Th17 differentiation but also functional heterogeneity. Hyperpolarized ΔΨ cells exhibit enhanced differentiation and cytokine production potential, but reduced actual cytokine secretion, likely due to exhaustion‐related functional impairment, whereas depolarized ΔΨ cells are more effective in triggering inflammatory signalling and cytokine release.

## 3 Discussion

Mitochondrial metabolism plays a crucial role in T cell activation, differentiation, and function [23], and exhibits distinct patterns in effector versus memory T cells [24]. In this study, we investigated how mitochondrial activity influences Th17 differentiation from naïve CD4^+^ T cells. Using an in vitro human Th17 differentiation model, we found that Th17 polarisation is accompanied by a metabolic shift—characterised by a reduced reliance on glycolysis and increased dependence on mitochondrial oxidative phosphorylation (OXPHOS). This metabolic reprogramming occurs early during differentiation, linking mitochondrial activity to Th17 commitment. We observed that hyperpolarized ΔΨ cells exhibited enhanced differentiation capacity and increased Th17 formation. However, this was also accompanied by elevated expression of exhaustion markers PD‐1 and TIGIT, suggesting that heightened mitochondrial activity simultaneously supports both Th17 differentiation and functional constraints, such as exhaustion.

Our first key observation was that Th17 cells exhibit significantly higher ΔΨ compared to non‐Th17 CD4^+^ T cells, indicating increased mitochondrial activity. This finding was further validated by XTT and CTG assays, which showed a metabolic distinction between hyperpolarised and depolarised ΔΨ cells. Importantly, while hyperpolarised ΔΨ cells are not exclusively Th17 cells, these cells exhibited enhanced differentiation capacity and a greater potential to form Th17 cells, supporting the notion that mitochondrial activity plays a pivotal role in Th17 differentiation.

Mitochondrial fission, fusion, and mitophagy are critical for supporting T cell activation, differentiation, and survival [25, 26]. While much is known about their roles in T cell biology, the specific contribution of mitochondrial dynamics during Th17 differentiation remains incompletely understood. Recent studies indicate that OXPHOS promotes early Th17 development by driving expression of the pioneer transcription factor BATF, which is essential for Th17 lineage commitment, and by facilitating TCR and mTOR signalling [27]. In our study, we observed that mitochondrial dynamics undergo distinct changes over time during Th17 differentiation. At the early stage (48 h), hyperpolarized ΔΨ cells exhibited increased expression of *TEFM*, a key regulator of mitochondrial transcription elongation [28], suggesting enhanced mitochondrial gene expression at this time point. By 96 h, however, Th17 cells exhibited downregulation of *DRP1* (a mediator of mitochondrial fission) and *MFN2* (a regulator of mitochondrial fusion) [29, 30], indicating a shift in mitochondrial dynamics that corresponds with the later stages of differentiation. These findings suggest that mitochondrial‐related genes are differentially regulated at distinct stages of Th17 differentiation, with early upregulation of mitochondrial activity associated with differentiation commitment, and later changes in mitochondrial dynamics possibly reflecting functional specialisation.

To further explore the metabolic profiles beyond mitochondrial activity, we employed the SCENITH assay to analyse the metabolic programmes of hyperpolarized ΔΨ and depolarized ΔΨ subsets. Our findings support the well‐established concept of metabolic flexibility in T cell differentiation [24], in which early activation relies on glycolysis, followed by a shift toward OXPHOS, FAO, and AAO capacities in later stages to sustain effector functions.

Our data build upon this concept by demonstrating that hyperpolarized ΔΨ cells exhibit a stronger reliance on glycolysis early in differentiation, potentially contributing to their enhanced Th17 differentiation. This glycolytic preference highlights the metabolic adaptability of T cells during activation, ensuring efficient energy production in inflammatory environments that characterise Th17‐driven autoimmune diseases and Th17 cell differentiation within the tumour microenvironment [31, 32]. In both autoimmunity and cancer, changes in the balance between Th17 and Treg cells play a key role in disease progression—with heightened Th17 activity driving autoimmune inflammation, and expanded Treg populations often seen in cancers like CLL, where they may suppress anti‐tumour immunity [33]. In healthy immunity, Th17 cells protect against extracellular bacteria and fungi, yet our healthy donor–derived Th17 model allows controlled analysis of differentiation and metabolic changes. Notably, Th17 cells play a dual role in the tumour microenvironment, promoting tumour progression via immunosuppressive and pro‐angiogenic pathways, yet also mediating anti‐tumour effects through Th1‐like conversion and IFN‐γ production [33, 34]. Considering that tumour sites impose distinct chemokine (e.g., CCL22, CCL28) and nutrient constraints compared with inflamed autoimmune tissues, integrating these contexts could broaden the translational relevance of our model to both malignancy progression and autoimmune disease.

Furthermore, our SCENITH data reveal a dynamic shift in mitochondrial activity during Th17 polarisation. At 48 h, hyperpolarised ΔΨ cells predominantly rely on glycolysis, likely to support the rapid energy production and biosynthetic requirements for differentiation. By 96 h, mitochondrial activity increases, indicating a transition toward OXPHOS, which supports Th17 formation, though the cells exhibit limited effector function due to increased exhaustion markers. This shift likely explains why hyperpolarised ΔΨ cells generate more Th17 cells compared to depolarised ΔΨ cells. OXPHOS has been implicated in long‐term T cell survival by preventing apoptosis, suggesting that metabolic adaptation contributes to the durability of Th17 responses [18].

In contrast, our FCCP data highlight that early mitochondrial activity is indispensable for Th17 differentiation. Adding FCCP at the onset of differentiation (0 h) significantly reduces Th17 yield, highlighting the critical role of mitochondrial function in initiating differentiation. However, when FCCP is added at 48 h, its inhibitory effect is less pronounced, suggesting that by this stage, differentiation is more robust and can tolerate mitochondrial disruption. These findings demonstrate the role of mitochondrial metabolism in regulating Th17 differentiation and function, with early mitochondrial activity being essential for initiation and later stages relying on metabolic flexibility to sustain differentiation and effector function.

To investigate how metabolic heterogeneity translates into functional differences, we assessed the activation states and signalling capacities of hyperpolarized ΔΨ and depolarized ΔΨ cells. While hyperpolarized ΔΨ cells exhibited increased expression of activation markers such as CD25 and CD69, their ability to trigger inflammatory signalling, measured by NF‐κB and AP‐1 activation in luciferase reporter fibroblasts, was surprisingly weaker compared to depolarized ΔΨ cells. These findings suggest that elevated activation marker expression in hyperpolarized ΔΨ cells does not necessarily correlate with stronger inflammatory signalling. Instead, our data imply that metabolic state influences not only the degree of T cell activation but also the capacity to drive inflammatory responses in the immune microenvironment. This highlights the complexity of metabolic programming in regulating intercellular communication, with hyperpolarized ΔΨ cells potentially driving more activation but exhibiting less efficient inflammatory signalling compared to depolarized ΔΨ cells.

Paradoxically, although hyperpolarized ΔΨ cells display elevated activation markers and higher intracellular cytokine production, their reduced actual cytokine secretion (e.g., IFN‐γ and GZMB) suggests the presence of intrinsic regulatory mechanisms that temper effector output. This paradox becomes more apparent when considering the link between heightened activation and cellular exhaustion. Hyperpolarized ΔΨ cells express elevated levels of exhaustion markers, including PD‐1 and TIGIT, which are associated with chronic activation and immune regulation [35]. Exhaustion, resulting from prolonged activation, impairs cellular function and serves as a protective mechanism to prevent excessive immune responses and maintain immune homeostasis [36]. The concurrent upregulation of PD‐1 and TIGIT is consistent with findings from chronic infection and cancer models, where sustained antigen exposure drives functional exhaustion. Importantly, studies in CD8^+^ T cells have shown that PD‐1 blockade can transiently restore effector function but often fails to reprogram exhausted cells into durable memory populations when antigen levels remain high, due to stable epigenetic constraints [37]. This raises the question of whether targeting PD‐1 pathways or mitochondrial function could produce lasting restoration of Th17 activity.

Addressing this will require in vivo validation in established autoimmune models such as EAE or collagen‐induced arthritis, enabling direct assessment of how checkpoint or metabolic modulation impacts Th17 persistence, functional polarisation, and disease progression. Specifically, reduced IL‐17A secretion can impair pathogen clearance or tissue repair, promoting persistent inflammation and tissue damage. This loss of functional Th17 cells results in an imbalance between immune activation and regulation, contributing to persistent inflammation, tissue damage, and disease progression. Recent studies suggest that targeting exhaustion markers like PD‐1 and TIGIT could restore Th17 function, enhance immune responses, and mitigate disease progression in these disorders [36, 38]. While our study does not directly assess therapeutic modulation of exhaustion, our findings suggest a potential link between mitochondrial activity and Th17 dysfunction. Future studies will be needed to determine whether targeting mitochondrial function or exhaustion pathways could restore Th17 effector capacity and influence autoimmune and cancer outcomes. In cancer immunotherapy, metabolic reprogramming, such as targeting mitochondrial dysfunction and PD‐1 pathways, has successfully restored the activity of exhausted T cells and improved their persistence and function [39, 40].

Our findings suggest that heightened mitochondrial activity in hyperpolarized ΔΨ cells may drive both increased activation and the development of exhaustion, ultimately impairing their ability to secrete cytokines effectively, despite their enhanced differentiation potential. This highlights a critical gap in our understanding of Th17 cell biology, specifically in how mitochondrial activity contributes to the induction of exhaustion during chronic inflammation. While targeting exhaustion markers like PD‐1 and TIGIT could be a promising therapeutic approach, our findings emphasise the need for further investigation into how mitochondrial function in Th17 cells can be modulated to restore immune responses and prevent pathogenic dysfunction. Given the growing evidence linking mitochondrial function to T cell exhaustion [41], future research into mitochondrial dynamics in Th17 cells during autoimmune diseases may uncover novel therapeutic targets for modulating immune responses and mitigating disease progression.

In summary, our findings highlight the critical role of early mitochondrial activity in shaping Th17 differentiation. Cells with heightened mitochondrial activity gradually decrease their reliance on glycolysis while increasing OXPHOS contribution over time, supporting both Th17 activation and differentiation. However, despite maintaining cytokine production potential, their actual cytokine secretion is impaired due to exhaustion. These results emphasise the importance of mitochondrial activity in balancing Th17 differentiation and functional persistence. We acknowledge the limitation of isolating pure RORγt^+^IL‐17A^+^ Th17 cells due to their low frequency and the effects of stimulation on mitochondrial measurements. Additionally, our study focused on the 48 and 96 h timepoints, which were chosen to represent early activation and later functional stages of Th17 cells. Including earlier timepoints, such as 24 h to capture initial gene activation during differentiation, as well as intermediate points like 72 h, could offer an overview picture of how mitochondrial regulation and exhaustion develop over time. Due to practical constraints, we limited our analysis to these two key stages but recognise that examining additional timepoints would be valuable for future research. Future studies should also employ improved sorting techniques and relevant in vivo models to better understand how mitochondrial activity regulates Th17 function and pathogenicity. Importantly, mitochondrial metabolism represents a promising target to modulate Th17‐driven immune responses, potentially leading to novel metabolic interventions for autoimmune diseases.

## 4 Methods

### 4.1 PBMC Isolation

Human peripheral blood mononuclear cells (PBMCs) (obtained from Sanquin, The Netherlands, project number: NVT 0397‐02) were isolated from buffy coats of healthy donors' peripheral blood by Ficoll Pacque PLUS density centrifugation according to the manufacturer's guidelines. After isolation, PBMCs were cryopreserved in 10% HPS (Gibco‐Invitrogen, Cat No. 16000‐044), 10% DMSO (MP Biomedicals, Cat No. 196055) in RPMI 1640 medium (Gibco‐Invitrogen, Cat No. 11875093) and stored in liquid nitrogen until further use.

### 4.2 Naïve CD4+ T Cell Isolation and Th17 Differentiation

To isolate naïve CD4+ T cells, cryopreserved PBMCs were thawed and washed twice using RPMI 1640 medium supplemented with 10% heat‐inactivated FCS + 100 U/mL penicillin, 100 mg/mL streptomycin. CD4+ naïve T cells were negatively selected using the Naive CD4+ T Cell Isolation Kit II, human (Miltenyi Biotec, 130‐094‐131) according to the manufacturer's protocol. After isolation, enriched naïve CD4+ T cells exhibited more than 95% purity and viability in the majority of the donors for experiments, as evaluated by flow cytometry staining for CD4, CD45RA, and viability marker 7‐AAD (Figure S3). The isolated naïve CD4+ T cells were cultured with XVIVO 15 medium (Lonza, cat02‐53Q) supplemented with 5% Cell‐Vive T‐NK Xeno‐Free Serum Substitute (BioLegend, 420502), 100 U/mL penicillin, 100 mg/mL streptomycin, and 100 mg/L pyruvate, at a density of 100 000 cells/well in 96‐well round‐bottom plates (Greiner). Cells were activated with precoated anti‐CD3 (clone OKT‐3, BioLegend) 5 μg/mL overnight at 4°C and anti‐CD28 0.25 μg/mL (clone 28.2, BioLegend) and with a cocktail containing a final concentration of anti‐human IFN‐γ 10 μg/mL (BioLegend, 506 533), anti‐human IL‐4 10 μg/mL (Biolegend, 500839), IL‐2 10 IU/mL (BioLegend, 589 102), TGF‐β1 1 ng/mL (ThermoFisher, 100–21‐10UG), IL‐6 30 ng/mL (Biolegend, 570802), IL‐23 10 ng/mL (BioLegend, 574 102), and IL‐1β 10 ng/mL (R&D system, 201‐LB) in complete XVIVO 15 medium to differentiate them into Th17 cells.

### 4.3 Antibody Staining and Flow Cytometry Analysis

Approximately 1 × 10^6^ cells per condition/sample were washed twice with PBS and labelled with ViaKrome 808 fixable viability dye (Beckman Coulter) at a dilution of 1.5:1000 in PBS for 30 min at 4°C in the dark. After washing, cells were optionally stained with antibodies targeting extracellular markers, fluorescently labelled, for 25 min at room temperature, protected from light, in 100 μL staining buffer (PBS + 1% BSA). BSA (bovine serum albumin) is used to block nonspecific binding sites on cells.

For intracellular staining, cells were fixed with 50 μL Foxp3 Transcription Factor Staining Buffer (ThermoFisher, 00‐5523‐00) for 25 min at room temperature, following the manufacturer's instructions. After fixation, cells were washed and stained with antibodies targeting intracellular markers for 20 min at room temperature in the dark.

To detect intracellular cytokines, cells were pre‐stimulated with 50 ng/mL phorbol 12‐myristate 13‐acetate (PMA, Merck, P1585‐1MG), 1 μg/mL ionomycin (Merck, I0634‐1MG), and 1 μg/mL Golgi Plug (BD Biosciences, 555 029) for 4 h prior to staining. Samples were acquired immediately after staining using a Beckman Coulter CytoFLEX LX 21‐colour flow cytometer. Flow cytometry data were analysed with Kaluza software version 2.1.3 (Beckman Coulter). Details of all antibodies used for extracellular and intracellular staining are provided *in* Tables S2 and S3.

### 4.4 Fluorescence‐Activated Cell Sorting

To generate cells with relatively high and low metabolic activity, naïve CD4^+^ T cells were cultured with a Th17 polarisation cocktail and stained with MitoTracker dye (ThermoFisher, M22426) at a dilution of 1:6000 in staining buffer (PBS + 1% BSA) for 30 min at 37°C. Cells were also stained with 7‐AAD (ThermoFisher, A1310) at a concentration of 1:1000 at approximately 48 and 96 h. After staining, cells were resuspended in staining buffer at a concentration of ~50 × 10^6^ cells/mL and sorted using a Cytek Aurora cytometer under high pressure (70 psi) with a 70 μm nozzle at a maximum flow rate setting of 6/11, maintained at 4°C.

For sorting, the top and bottom 20% of cells with hyperpolarized ΔΨ and depolarized ΔΨ based on MFI were collected into polypropylene tubes containing 2 mL of XVIVO 15 medium, supplemented with 5% Cell‐Vive T‐NK Xeno‐Free Serum Substitute, 100 mg/L pyruvate, and 1% Penicillin–Streptomycin. Post‐sort, both hyperpolarized ΔΨ cells and depolarized ΔΨ cell populations were assessed for viability (approximately 96%) and purity. Acquisition was performed using the same instrument layout. An overview of the gating strategy is provided in Figure S4.

### 4.5 XTT Assay

To evaluate NADH‐dependent metabolic activity in sorted cells, the XTT Cell Viability Kit (Cell Signalling, #9095) was used according to the manufacturer's instructions. Sorted hyperpolarized ΔΨ and depolarized ΔΨ cells were washed in RPMI 1640 Medium (no phenol red, Gibco, 11835030) and resuspended at a final concentration of 250 000 cells/mL. A total of 100 μL per well was seeded into a 96‐well round‐bottom plate. Then, 50 μL of XTT working solution was added to each well and mixed. Absorbance was measured every 15 min from 0 to 120 min using a Clariostar (BMG LABTECH) microplate reader.

Four technical replicates were performed per condition, and the average value was used for subsequent analysis. Both negative and positive controls were included to ensure assay validity. The negative control consisted of media without cells, while the positive control was generated using untreated Jurkat cells with known metabolic activity.

### 4.6 CellTiter‐Glo Assay

To assess ATP production in sorted cells, the CellTiter‐Glo Luminescent Cell Viability Assay (Promega, G9241) was performed following the manufacturer's instructions. Hyperpolarized ΔΨ and depolarized ΔΨ cells were washed in RPMI (no phenol red) and resuspended at 125,000 cells/mL. 50 μL of cell suspension was added to a 96‐well flat‐bottom plate, followed by 50 μL of CellTiter‐Glo reagent. The plate was covered with aluminium foil to protect it from light and incubated for 10 min at room temperature. Luminescence was then measured using a Clariostar (BMG LABTECH).

Each condition was tested in four technical replicates, and mean values were used in the analysis. Values were corrected by subtracting background luminescence from medium‐only wells (negative control). A positive control using metabolically active Jurkat cells was included to verify assay sensitivity.

### 4.7 RNA Isolation and Quantitative Real‐Time PCR

Sorted cells were collected at 48 and 96 h after FACS, and RNA was extracted using TRIzol reagent (Sigma‐Aldrich, Saint Louis, MO, USA). For every 500,000 cells, 500 μL of TRIzol was added, followed by cell lysis. Subsequently, 100 μL of chloroform was added, and the samples were centrifuged at 11,600×*g* for 15 min at 4°C. The aqueous phase was carefully collected. To precipitate RNA, a 1:1 mixture of isopropanol and 1:10 sodium acetate buffer (3 M; Sigma‐Aldrich) was added, and samples were incubated overnight at −20°C. The samples were then centrifuged at 11,600×*g* for 30 min at 4°C. The RNA pellet was washed with 75% ethanol and reconstituted in RNase‐free water.

To remove residual genomic DNA, RNA samples were treated with DNase I (Roche, Basel, Switzerland) according to the manufacturer's instructions. cDNA synthesis was performed using standard protocols. Quantitative real‐time PCR (qPCR) was carried out using Power SYBR Green PCR Master Mix (Applied Biosystems, 4 368 708, Waltham, MA, USA) on an Applied Biosystems QuantStudio 1 Real‐Time PCR System. Primer information used in the qPCR is listed in Table S4. All primers were validated for amplification efficiency prior to use. The selection of reference genes is provided in Figure S5.

Gene expression was calculated relative to the housekeeping genes *HSDNA*, *PL37A*, and *RPS27A*, and is presented as −ΔCt values. The −ΔCt values were calculated as follows:

−∆CT=AverageCTof3reference genes−CTvalue of the gene of interest.

### 4.8 SCENITH

#### 4.8.1 SCENITH Assay for Metabolic Profiling of Th17‐Differentiated CD4 ^*+*^ T Cells

SCENITH was performed as described by Argüello et al. [3], leveraging the close relationship between protein synthesis and ATP levels to assess cellular metabolic dependencies. This method evaluates the impact of metabolic inhibitors on puromycin incorporation during protein translation, providing insights into glycolytic and mitochondrial contributions via flow cytometry.

#### 4.8.2 Cell Preparation and Culture Conditions

Naïve CD4^+^ T cells were isolated and plated at a density of 1 × 10^*6*^ cells/mL in 100 μL per well within 96‐well round‐bottom plates, under Th17‐polarising conditions. Cells were cultured in complete X‐VIVO medium devoid of pyruvate to prevent interference with the SCENITH assay's metabolic readouts. For each condition, five wells were prepared for the 48‐h time point and three wells for the 96‐h time point. Cells from respective wells were pooled prior to flow cytometry analysis. Experiments encompassed samples from 11 individual donors, with data from each pooled condition utilised for subsequent analyses.

#### 4.8.3 Metabolic Inhibitor Treatments

At designated time points, cells underwent treatment with the following metabolic inhibitors for 15 min:

- 2‐Deoxy‐D‐glucose (2‐DG) at a final concentration of 100 mM
- Oligomycin (O) at a final concentration of 1 μM
- A combination of 2‐DG and Oligomycin (DGO)
- Control (medium only)

To serve as a negative control, 2 μg/mL of the translation initiation inhibitor Harringtonine was added 15 min prior to puromycin administration. Primary antibody information is listed in Table S5.

#### 4.8.4 Puromycin Incorporation and Staining

Subsequent to inhibitor treatments, puromycin was introduced at a final concentration of 10 μg/mL for an additional 15 min. Cells were then washed with cold PBS and stained with a combination of Fc receptor blockade and ViaKrome 808 viability dye (Beckman Coulter, Pasadena, CA, USA) for 30 min. Following staining, cells were fixed and permeabilised using the FOXP3 Fixation/Permeabilisation Buffer Set (Thermo Fisher eBioscience), in accordance with the manufacturer's instructions.

Intracellular staining for puromycin was performed using Alexa Fluor 488‐conjugated anti‐puromycin antibody (clone 2A4, BioLegend, 381 506). Cells were incubated for 1 h at 4°C in a 1:10 dilution of the permeabilisation buffer. Data acquisition was executed on a CytoFlex LX flow cytometer (Beckman Coulter).

#### 4.8.5 Data Analysis

SCENITH calculation was performed as described by Argüello et al., utilising median fluorescence intensity (MFI) values of puromycin incorporation to determine metabolic dependencies and capacities.

(1) CONTROL=gMFI of anti−Puro−Fluorochrome upon Control treatment

(2) 2DG=gMFI of anti−Puro−Fluorochrome upon2DGtreatment

(3) O=gMFI of anti−Puro−Fluorochrome upon Oligomycin treatment

(4) $$\mathrm{DGO}=\text{gMFI of anti}-\text{Puro}-\text{Fluorochrome upon}\;\left(2-\mathrm{DG}+\text{Oligomycin}\right)\;\text{treatment}$$

(5) H=gMFI of anti−Puro Fluorochrome upon Harringtonine treatment

(6) $$\text{Glucose dependence}=\frac{100\times \left(\text{control}-2-\mathrm{DG}\right)}{\text{Control}-\mathrm{DGO}}$$

(7) $$\text{Mitochondrial dependence}=\frac{100\times \left(\text{control}-O\right)}{\text{Control}-\mathrm{DGO}}$$

(8) Glycolytic capacity=100−Mitochondrial dependence

(9) FAAandAAO=100−Glucose dependence

(10) $$\begin{array}{c} \Delta \Psi \;\text{Depolarized}\;\text{cells}=\text{approximately the lowest}\;20\%\text{of anti}\\ \;-\text{MitoTracker Deep}\;\mathrm{Red}-\text{Fluorochrome} \end{array}$$

(11) $$\begin{array}{c} \Delta \Psi \;\text{Hyperpolarized cells}=\text{approximately}\;\text{highest}\;20\%\text{of anti}\\ \;-\text{MitoTracker Deep}\;\mathrm{Red}-\text{Fluorochrome} \end{array}$$

### 4.9 Cytokine Measurements

Human cytokines in the culture supernatants were quantified using the Luminex platform. Cell culture supernatants were collected, centrifuged at 300 g for 10 min, and then transferred to new 500 μL Eppendorf tubes, which were stored at −20°C. Cytokine levels were measured using the Human Luminex Discovery Assay (Bio‐Techne, LXSAHM‐06) according to the manufacturer's instructions. Sample analysis was performed using BioPlex Manager 4 software (Bio‐Rad Laboratories, Hercules, CA, USA).

### 4.10 Co‐Culture Reporters' Luciferase Assay

The activation of JAK‐STAT (SIE), MAPK (AP‐1), and NF‐κB (NF‐κB) signalling pathways was evaluated using reporter constructs cloned into primary dermal myofibroblasts, originally obtained from Neefjes et al. [42]. Reporter fibroblasts were seeded at a density of 150,000 cells per well. IL‐6 was used as a positive control for the JAK–STAT (SIE) reporter, PMA + Ionomycin for the MAPK (AP‐1) reporter, and TNF‐α for the NF‐κB reporter. After 24 h of co‐culture with a 1:1 ratio of hyperpolarized ΔΨ cells and depolarized ΔΨ cells, the cells were collected and centrifuged. The resulting pellet was resuspended in 150 μL of Assay Buffer containing a 50× substrate from the Nano‐Glo Luciferase Assay Kit (Promega). Luciferase activity was measured using a ClarioStar plate reader (BMG Labtech), with emission recorded at 590 nm.

### 4.11 Statistical Analysis

Statistical analyses were performed using GraphPad Prism software. First, the normal distribution of the data was assessed. If the data followed a normal distribution, paired t‐tests were used to compare two matched experimental groups from independent donors. If the data did not follow a normal distribution, Wilcoxon signed‐rank tests were used for comparisons. Data for Figures 1G–I and 2H show the mean of multiple donors, and error bars represent the mean ± SD.

For qPCR data, the expression levels of mitochondrial dynamic genes were analysed within hyperpolarized ΔΨ cells and depolarized ΔΨ cells from 6 independent donors. Paired analyses were performed for each donor, and *Z* scores were calculated for each gene. The *Z* scores were then plotted in a heatmap to visualise gene expression patterns across conditions. *Z* scores were calculated using the following formula:

$$Z=\frac{\left(X-\mu \right)}{\sigma }$$

where *X* is the gene expression value, *μ* is the mean gene expression across all conditions, and *σ* is the standard deviation of the gene expression values.

For flow cytometry analysis, gMFI values and percentages were determined using Kaluza software (Beckman Coulter) and were used for subsequent statistical analysis. Dimensionality reduction, viSNE analysis, and plots were processed via Cytobank (Beckman Coulter). *p* values are indicated by *p < 0.05, **p < 0.01, ***p < 0.001, and ****p < 0.0001, where statistically significant differences were observed.

## Disclosure

**Policy on Using Generative** AI **and** AI **‐Assisted Technologies**: AI‐assisted technologies (e.g., grammar and language editing tools) were used to correct grammar and spelling errors in the manuscript. No AI tools were used to generate scientific content, perform data interpretation, or draw conclusions.

## Conflicts of Interest

The authors declare no conflicts interest.

## Supporting information


**Figure S1:** Flow Cytometry Gating Strategy for Th17 cells. The gating strategy for identifying Th17 cells is as follows: First, debris and doublets are excluded using FSC vs. SSC and FSC‐A vs. FSC‐H gates. Live cells are then selected based on side scatter and Viakrom808 characteristics. RORγt+ cells are identified, followed by gating for IL‐17A+ cells. The final population of RORγt+ IL‐17A+ cells, defined as Th17 cells, is identified based on the appropriate isotype controls and fluorescence minus one (FMO) or non‐staining controls for gating validation.
**Figure S2:** Three Th17 differentiation cocktails result in different cytokine and transcription factor profiles. To further investigate the differentiation profiles across the three Th17 cocktails, we applied semi‐supervised flow cytometric analysis using vi‐SNE software (Cytobank). The analysis was performed with an equal input of 8700 cells per cocktail sample. In addition to IL‐17A and IFN‐gamma, we measured the expression of other T helper subset‐related transcription factors and cytokines, including IL‐4, T‐bet, FoxP3, IL10 and so on. The results highlight differences in cytokine and transcription factor expression across the cocktails, providing a more comprehensive view of the differentiation outcomes and confirming the specificity of the cocktails in driving distinct T helper subset profiles.
**Figure** S**3**. Naïve CD4 T cell isolation kit purity check Flow cytometry analysis was performed to assess the purity of naïve CD4 T cells following isolation using the Miltenyi Biotec negative selection kit. Initially, CD45RO‐FITC staining was used to confirm the absence of memory T cells, with almost all cells being CD45RO‐negative. Subsequently, CD4 SPARK‐UV staining was applied to confirm that over 90% of the isolated cells were CD4+ T cells, ensuring the high purity of the isolated naïve CD4 T cell population.
**Figure S4:** Flow Cytometry Sorting Strategy for Isolation hyperpolarized ΔΨ cells and depolarized ΔΨ cells populations. The flow cytometry sorting process involved sequential gating to ensure high purity of the sorted population. First, debris was excluded, followed by singlet selection to isolate individual cells. Viability was then assessed to ensure only live cells were included in subsequent analysis. hyperpolarized ΔΨ and depolarized ΔΨ populations were sorted based on MitoTracker Deep Red staining, with approximately 20% of the cells classified hyperpolarized ΔΨ cells. After sorting, cell purity, viability, and cell number were reassessed to confirm the integrity and quality of the sorted population.
**Figure S5:** Gene selection for mitochondrial gene expression analysis.

**Table S1:** Th17 polarisation cocktails.
**Table S2:** List of antibodies used for cell surface flow cytometry staining.
**Table S3:** List of antibodies used for intracellular flow cytometry staining.
**Table S4:** List of primers used for qPCR.
**Table S5:** List of SCENITH Reagents.

## Data Availability

The data that support the findings of this study are available from the corresponding author upon reasonable request.
