# Supplementary material for: Mitochondrial Activity Regulates Human T Helper 17 Differentiation and Function
Source: Immunology. 2025 Sep 17;177(1):185–98. doi: 10.1111/imm.70037 (PMC12665803; doi:10.1111/imm.70037)
Supplement: Supplementary file 1 — Figure S1: Flow Cytometry Gating Strategy for Th17 cells. The gating strategy for identifying Th17 cells is as follows: First, debris and doublets are excluded using FSC vs. SSC and FSC‐A vs. FSC‐H gates. Live cells are then selected based on side scatter and Viakrom808 characteristics. RORγt+ cells are identified, followed by gating for IL‐17A+ cells. The final population of RORγt+ IL‐17A+ cells, defined as Th17 cells, is identified based on the appropriate isotype controls and fluorescence minus one (FMO) or non‐staining controls for gating validation. Figure S2: Three Th17 differentiation cocktails result in different cytokine and transcription factor profiles. To further investigate the differentiation profiles across the three Th17 cocktails, we applied semi‐supervised flow cytometric analysis using vi‐SNE software (Cytobank). The analysis was performed with an equal input of 8700 cells per cocktail sample. In addition to IL‐17A and IFN‐gamma, we measured the expression of other T helper subset‐related transcription factors and cytokines, including IL‐4, T‐bet, FoxP3, IL10 and so on. The results highlight differences in cytokine and transcription factor expression across the cocktails, providing a more comprehensive view of the differentiation outcomes and confirming the specificity of the cocktails in driving distinct T helper subset profiles. Figure S3. Naïve CD4 T cell isolation kit purity check Flow cytometry analysis was performed to assess the purity of naïve CD4 T cells following isolation using the Miltenyi Biotec negative selection kit. Initially, CD45RO‐FITC staining was used to confirm the absence of memory T cells, with almost all cells being CD45RO‐negative. Subsequently, CD4 SPARK‐UV staining was applied to confirm that over 90% of the isolated cells were CD4+ T cells, ensuring the high purity of the isolated naïve CD4 T cell population. Figure S4: Flow Cytometry Sorting Strategy for Isolation hyperpolarized ΔΨ cells and depolarized ΔΨ cells popul [file IMM-177-185-s001.docx]

### **Supplementary Figures**

**
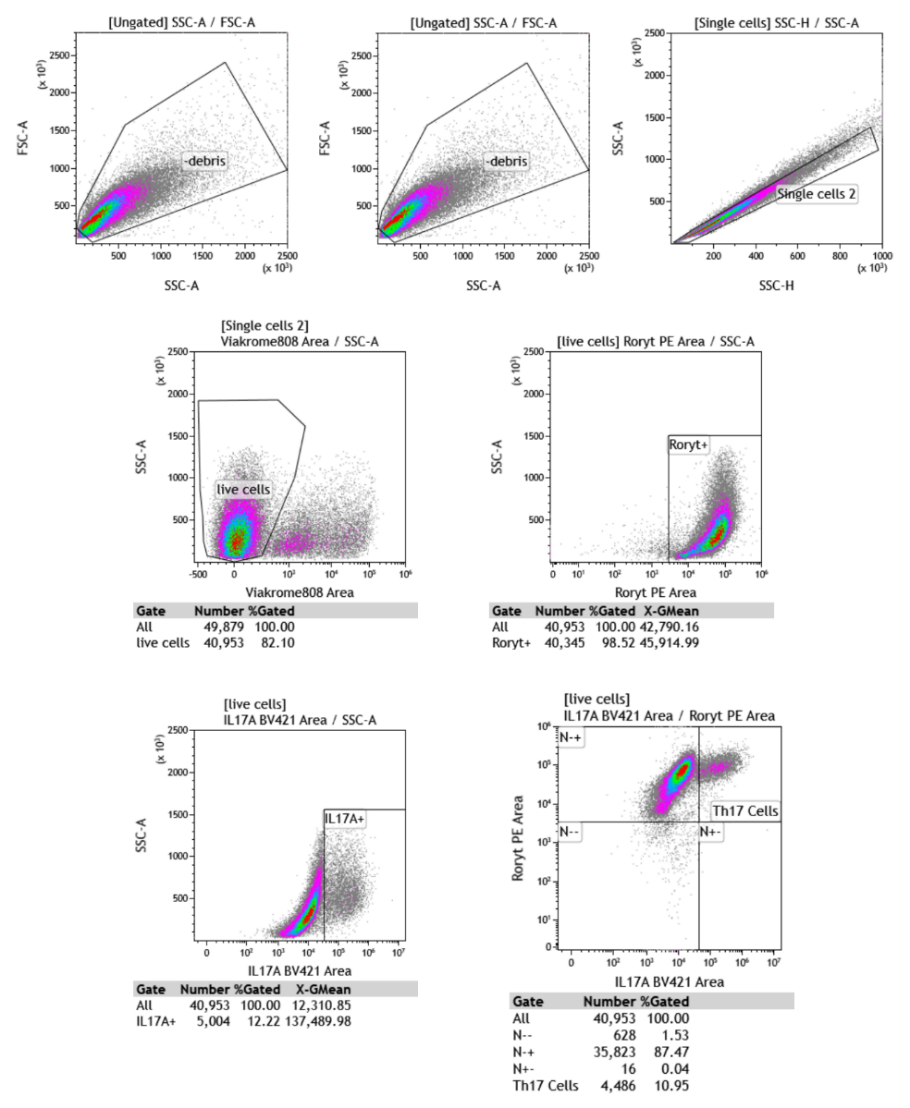
**

**Supplementary figure 1 Flow Cytometry Gating Strategy for Th17 cells.** The gating strategy for identifying Th17 cells is as follows: First, debris and doublets are excluded using FSC vs. SSC and FSC-A vs. FSC-H gates. Live cells are then selected based on side scatter and Viakrom808 characteristics. RORγt+ cells are identified, followed by gating for IL-17A+ cells. The final population of RORγt+ IL-17A+ cells, defined as Th17 cells, is identified based on the appropriate isotype controls and fluorescence minus one (FMO) or non-staining controls for gating validation.


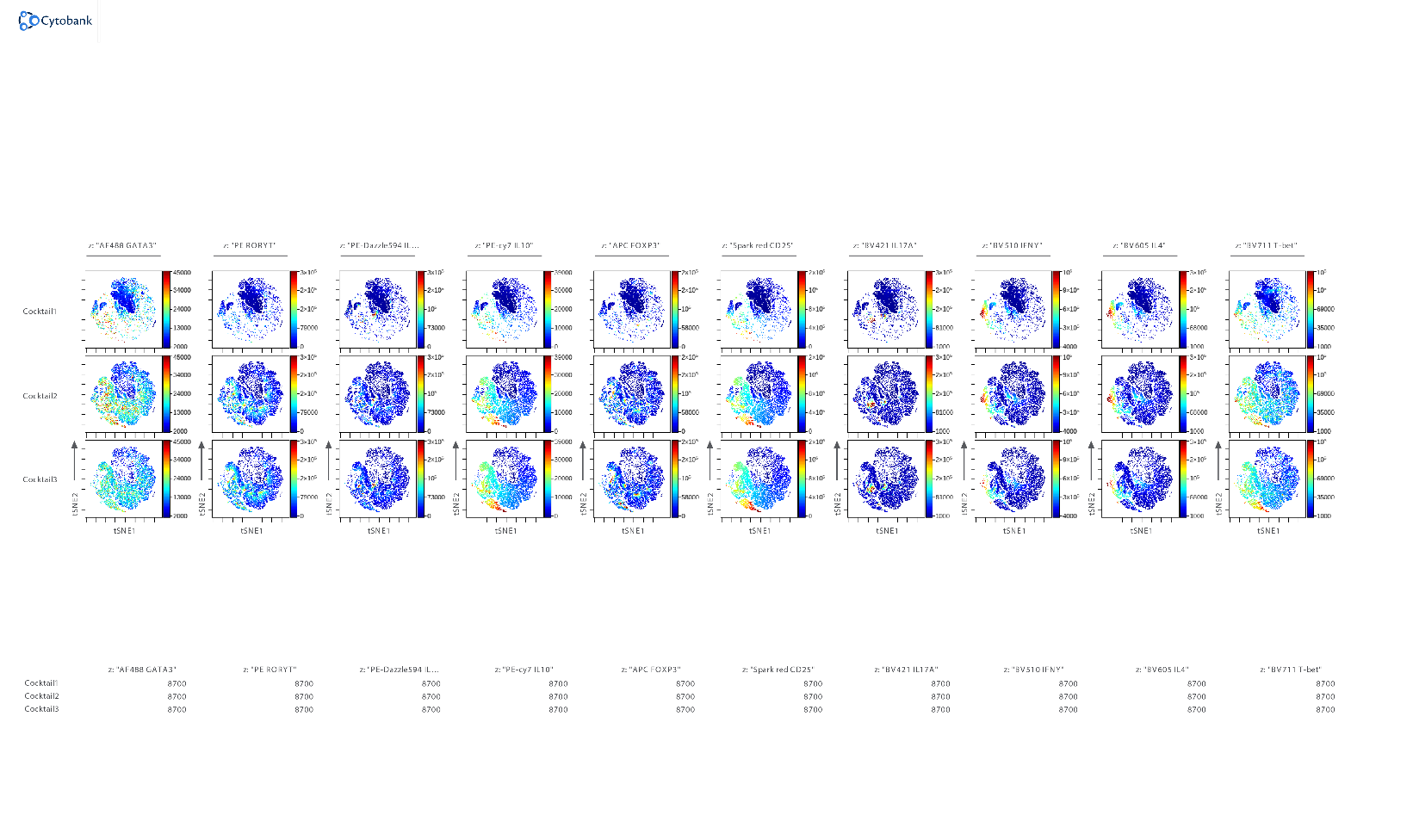


**Supplementary figure 2 Three Th17 differentiation cocktails result in different cytokine and transcription factor profiles.** To further investigate the differentiation profiles across the three Th17 cocktails, we applied semi-supervised flow cytometric analysis using vi-SNE software (Cytobank). The analysis was performed with an equal input of 8700 cells per cocktail sample. In addition to IL-17A and IFN-gamma, we measured the expression of other T helper subset-related transcription factors and cytokines, including IL-4, T-bet, FoxP3, IL10 and so on. The results highlight differences in cytokine and transcription factor expression across the cocktails, providing a more comprehensive view of the differentiation outcomes and confirming the specificity of the cocktails in driving distinct T helper subset profiles.

**
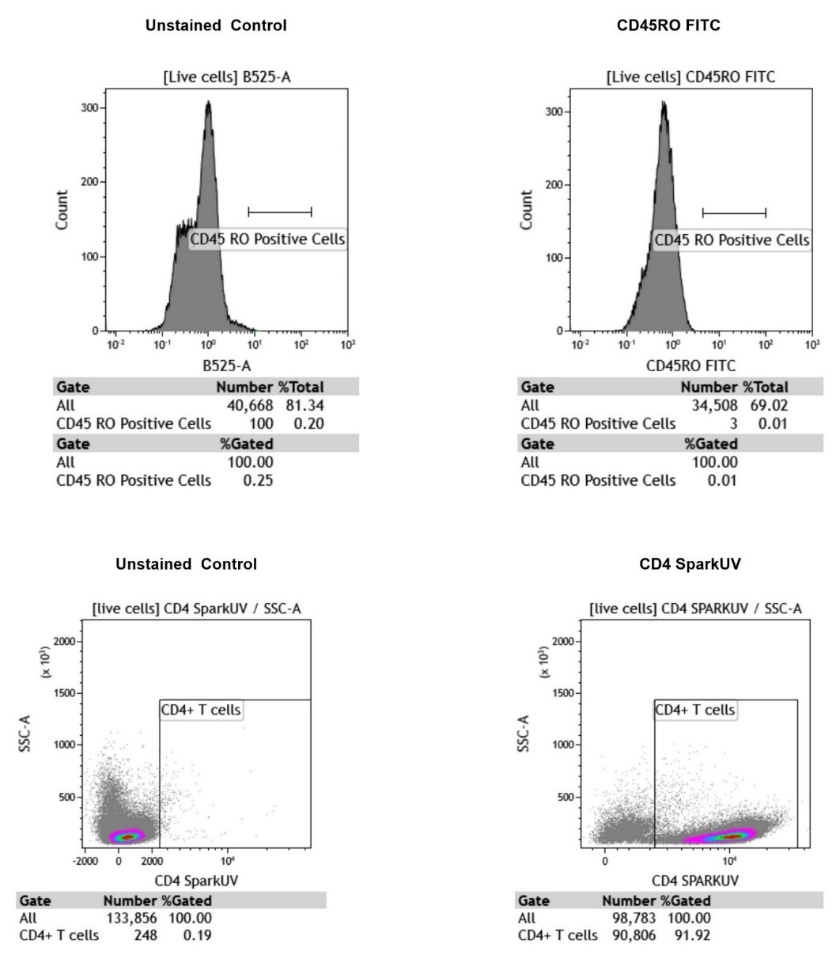
**

**Supplementary figure** **3 Naïve CD4 T cell isolation kit purity check** Flow cytometry analysis was performed to assess the purity of naïve CD4 T cells following isolation using the Miltenyi Biotec negative selection kit. Initially, CD45RO-FITC staining was used to confirm the absence of memory T cells, with almost all cells being CD45RO-negative. Subsequently, CD4 SPARK-UV staining was applied to confirm that over 90% of the isolated cells were CD4+ T cells, ensuring the high purity of the isolated naïve CD4 T cell population.

**
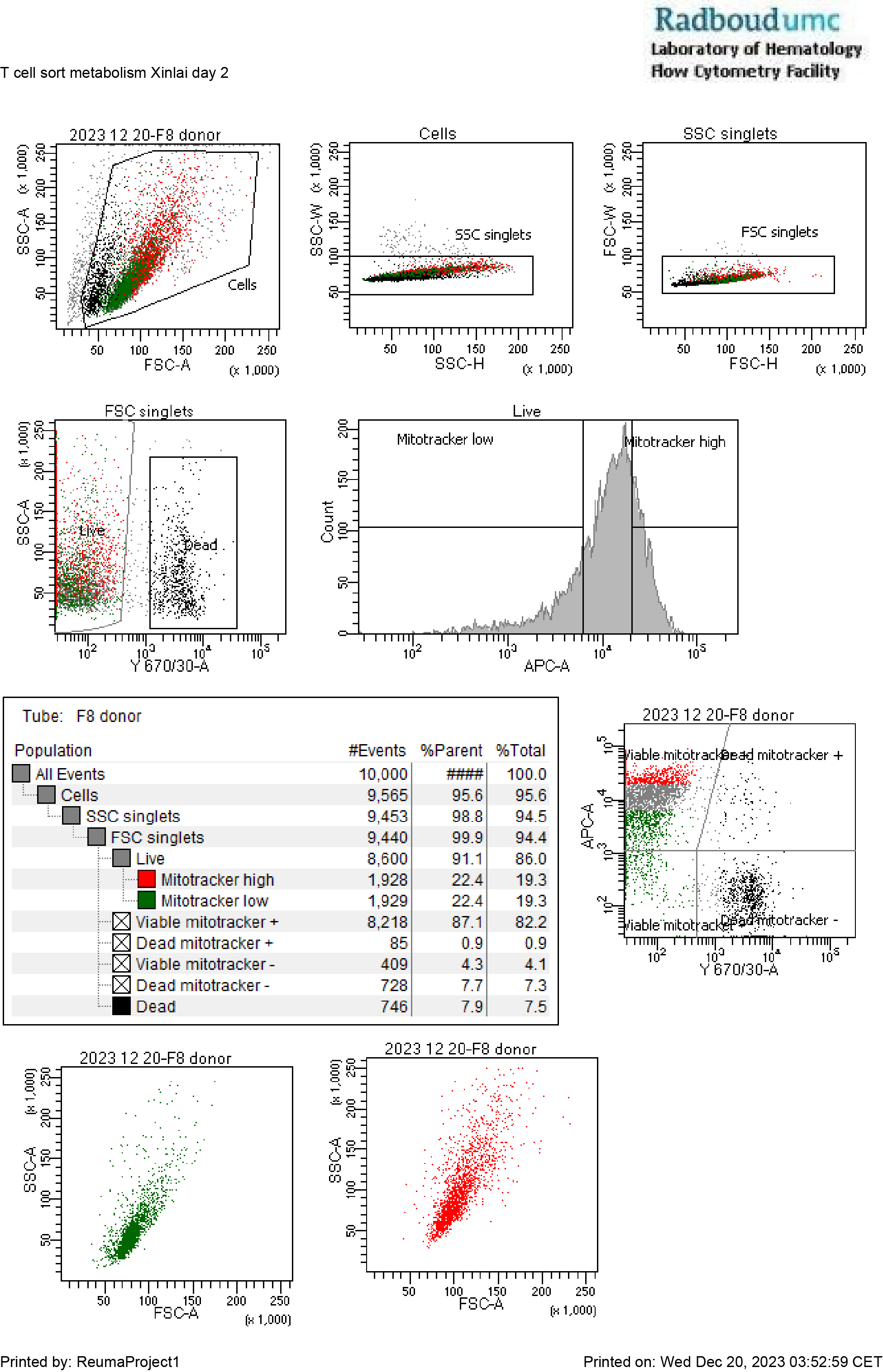
**

**Supplementary figure 4 Flow Cytometry Sorting Strategy for Isolation hyperpolarized ΔΨ cells and depolarized ΔΨ cells.** PopulationsThe flow cytometry sorting process involved sequential gating to ensure high purity of the sorted population. First, debris was excluded, followed by singlet selection to isolate individual cells. Viability was then assessed to ensure only live cells were included in subsequent analysis. **hyperpolarized ΔΨ and depolarized ΔΨ** populations were sorted based on MitoTracker Deep Red staining, with approximately 20% of the cells classified **hyperpolarized ΔΨ cells**. After sorting, cell purity, viability, and cell number were reassessed to confirm the integrity and quality of the sorted population.

**
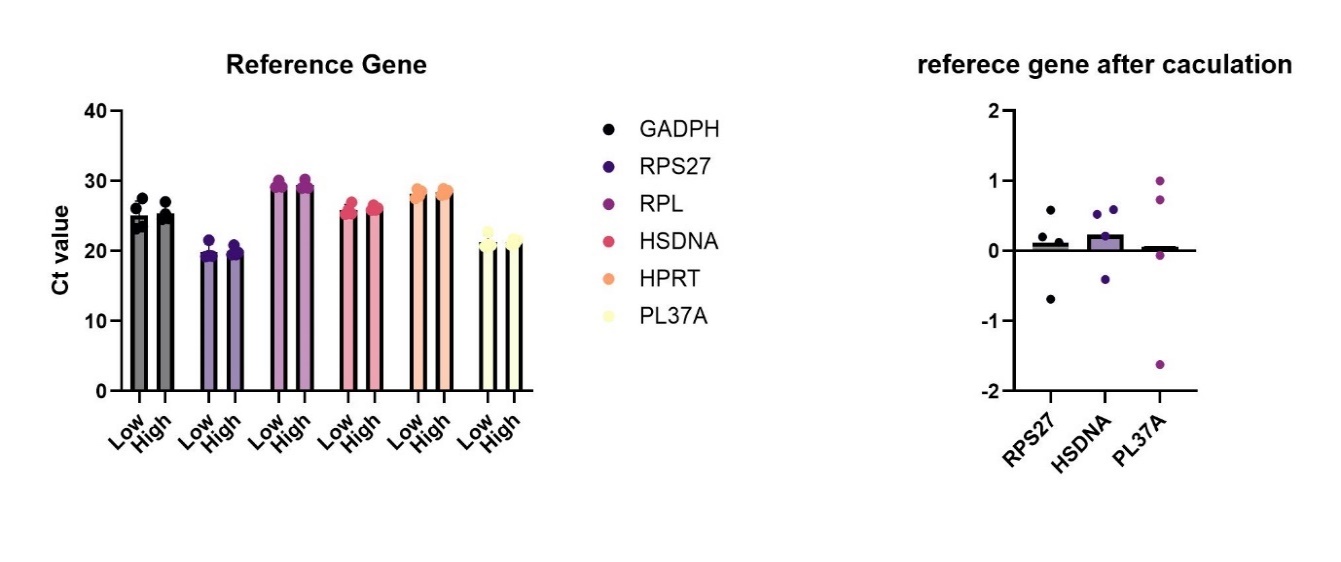
**

**Supplementary figure 5 Gene selection for mitochondrial gene expression analysis.**

To ensure the reliability of gene expression normalization in mitochondrial morphology and activity-related gene measurements (Figure 2F), reference genes with stable expression across distinct mitochondrial populations were validated. Naïve CD4+ T cells cultured in our Th17 differentiation cocktail for 48 hours were sorted into ΔΨm high and low populations based on MitoTracker Deep Red staining. The Ct values for six commonly used reference genes (GADPH, RPS27, RPL, HSDNA, HPRT, and PL37A) were assessed in three individual donors. As shown in the graph, no significant differences in Ct values were observed between ΔΨm high and low populations, confirming the stable expression of these reference genes. Based on these findings, RPS27, HSDNA, and PL37A were selected as the reference genes for subsequent qPCR analysis in Figure 2F, with the goal of achieving Ct values below 30 to ensure reliable normalization of mitochondrial-related gene expression.
