# Supplementary material for: Mitochondrial Activity Regulates Human T Helper 17 Differentiation and Function
Source: Immunology. 2025 Sep 17;177(1):185–98. doi: 10.1111/imm.70037 (PMC12665803; doi:10.1111/imm.70037)
Supplement: Supplementary file 2 — Table S1: Th17 polarisation cocktails. Table S2: List of antibodies used for cell surface flow cytometry staining. Table S3: List of antibodies used for intracellular flow cytometry staining. Table S4: List of primers used for qPCR. Table S5: List of SCENITH Reagents. [file IMM-177-185-s002.docx]

### **Supplementary Tables**

*Supplementary Table 1 Th17 polarization cocktails*

| *Cytokines/ reagents* | *cocktail 3*  *our cocktail* | *Additional cocktail 1* | *Additional cocktail 2* | *Supplier* | *Identifier* |
| --- | --- | --- | --- | --- | --- |
| CD3 | 5 µg/ml | 5 µg/ml | 5 µg/ml | BioLegend | *317326* |
| CD28 | 0.25 µg/ml | *-* | 2.5 µg/ml | BioLegend | *302934* |
| IL-1β | 10 ng/ml | 50 ng/ml | 10 ng/ml | *R&D* | *201-LB* |
| IL-2 | 10 U/ml | *-* | 10 U/ml | BioLegend | *589104* |
| TGF-β | 1 ng/ml | *-* | 1 ng/ml | Pepro Tech | *100-21c* |
| IL-6 | 30 ng/ml | *-* | 10 ng/ml | BioLegend | *570804* |
| IL-23 | 10 ng/ml | 50 ng/ml | *-* | BioLegend | *574102* |
| IL-21 | *-* | *-* | 20 ng/ml | BioLegend | *571202* |
| Anti-IFN-γ | 10 µg/ml | 10 µg/ml | 10 µg/ml | BioLegend | *506533* |
| Anti-IL-4 | 10 µg/ml | 10 µg/ml | 10 µg/ml | BioLegend | *500839* |

*Supplementary Table 2*: *List of antibodies used for cell surface flow cytometry staining*

| Antigen | Clone | Fluorochrome | Supplier | Identifier |
| --- | --- | --- | --- | --- |
| CD4 | SK3 | SPARK UV | BioLegend | 344685 |
| CD45R0 | UCHL1 | FITC | BioLegend | 983110 |
| CD25 | M-A251 | PE/CYANINE7 or SPARK UV | BioLegend | 356107/356157 |
| CD69 | FN50 | PE/DAZZLE594 | BioLegend | 310941 |
| PD-1 | EH12.2H7 | Brilliant Violet 421 | BioLegend | 329919 |
| TIGIT | VSTM3 | Brilliant Violet 785 | BioLegend | 372735 |

*Supplementary Table 3: List of antibodies used for intracellular flow cytometry staining.*

| Antigen | Clone | Fluorochrome | Supplier | Identifier |
| --- | --- | --- | --- | --- |
| RORyt | 12-6988-82 | PE | INVITROGEN | 12-6988-82 |
| IL-17A | BL168 | \| Brilliant Violet 421 \| \| --- \| \|  \| | BioLegend | 512322 |
| IFN-y | 4S.B3 | Brilliant Violet 510 | BioLegend | 502544 |
| Granzyme B | GB11 | FITC | BioLegend | 515403 |
| Anti puromycin | 2A4 | ALEXA FLUOR 488 | BioLegend | 381505 |
| Gata3 | 16E10A23 | ALEXA FLUOR 488 | BioLegend | 653807 |
| IL-22 | 2G12A41 | PEDazzle594 | BioLegend | 366715 |
| IL-10 | JES3-9D7 | PE-Cy7 | BioLegend | 501419 |
| Foxp3 | PCH101 | APC | INVITROGEN | 17-4776-42 |
| IL-4 | MP4-25D2 | PE/Dazzle594 | BioLegend | 500831 |
| T-bet | 4B10 | Brilliant Violet711 | BioLegend | 644819 |
| IL-6 | MQ2-13A5 | Pacific Blue | BioLegend | 501113 |
| IL-21 | eBIO3A3-N2 | PE | eBioscience | 12-7219-42 |
| ViaKrome 808 |  |  | Beckman Coulter | C36628 |

*Supplementary Table 4: List of primers used for qPCR.*

| Gene | Forward primer sequence (5’-3’) | Reverse primer sequence (3’-5’) |
| --- | --- | --- |
| *HSDNA* | TGGAGCTGCAGAACCTGATG | TGTAGTCTTCCCTGGCATGC |
| *PL37A* | ATTGAAATCAGCCAGCACG | AGGAACCACAGTGCCAGAT |
| *RPS27a* | TGGCTGTCCTGAAATATTATAAGGT | CCCCAGCACCACATTCATCA |
| *OPA1* | ACGTCTTTTGTCCAGCCTCT | GGTTAAAGCGCCCGTAACAT |
| *MFN1* | CCTGGCATCCAGGAGTTAGA | TGGTTCCAGCAATGCGATTT |
| *MFN2* | TGCAGGTGTAAGGGACGATT | GAGGCTCTGCAAATGGGATG |
| *DRP1* | CAAAGCAGTTTGCCTGTGGA | TCTTGGAGGACTATGGCAGC |
| *FIS1* | CCAAATCCTGAAGGAGACGC | GCTGAAGGCCACAGAGGATA |
| *TEFM* | TGAGAAAGCTCCTCAAACCAGAC | CAGTCCAGCACTGTCAACTTACG |
| *POLRMT* | GTCAACACCTCCAAGCTGCTC | GCTGCTTCTCAAAGAGGCACTG |
| *TFB2M* | GGGAAAACCAAGTAGACCTCCAC | TTTCGAGCGCAACCACTTTGGC |
| *NRF1* | GGCAACAGTAGCCACATTGGCT | GTCGTCTGGATGGTCATCTCAC |
| *GOT1* | CAACTGGGATTGACCCAACT | GGA ACA GAA ACC GGT GCT T |
| *RORC* | TGAGAAGGACAGGGAGCCAA | CCACAGATTTTGCAAGGGATCA |
| *IL17A* | TTGATTGGAAGAAACAACGATGA | CTCAGCAGCAGTAGCAGTGACA |

*Supplementary Table 5*. List of SCENITH Reagents.

| Chemicals | Supplier | Identifier |
| --- | --- | --- |
| Oligomycin A | Merck | 75351-5Mg |
| 2-Deoxy-D-glucose | Sanbio | 14325-1 |
| Harringtonine | Sanbio | 15361-1 |
